# When Grades Are High but Self-Efficacy Is Low: Unpacking the Confidence Gap Between Girls and Boys in Mathematics

**DOI:** 10.3389/fpsyg.2020.552355

**Published:** 2020-10-07

**Authors:** Lysann Zander, Elisabeth Höhne, Sophie Harms, Maximilian Pfost, Matthew J. Hornsey

**Affiliations:** ^1^Division of Empirical Educational Research, Institute of Education, Leibniz Universität Hannover, Hanover, Germany; ^2^Division of School and Teaching Research, Department of Educational Science and Psychology, Freie Universität Berlin, Berlin, Germany; ^3^Department of Educational Research, Institute of Education, Otto-Friedrich-Universität Bamberg, Bavaria, Germany; ^4^University of Queensland Business School, Brisbane, QLD, Australia

**Keywords:** self-efficacy beliefs, gender, mathematics, STEM, sources of self-efficacy

## Abstract

Girls have much lower mathematics self-efficacy than boys, a likely contributor to the underrepresentation of women in STEM. To help explain this gender confidence gap, we examined predictors of mathematics self-efficacy in a sample of 1,007 9th graders aged 13–18 years (54.2% girls). Participants completed a standardized math test, after which they rated three indices of mastery: an affective component (state self-esteem), a meta-cognitive component (self-enhancement), and their prior math grade. Despite having similar grades, girls reported lower mathematics self-efficacy and state self-esteem, and were less likely than boys to self-enhance in terms of performance. Multilevel multiple-group regression analyses showed that the affective mastery component explained girls’ self-efficacy while cognitive self-enhancement explained boys’. Yet, a chi-square test showed that both constructs were equally relevant in the prediction of girls’ and boys’ self-efficacy. Measures of interpersonal sources of self-efficacy were not predictive of self-efficacy after taking the other dimensions into account. Results suggest that boys are advantaged in their development of mathematics self-efficacy beliefs, partly due to more positive feelings and more cognitive self-enhancement following test situations.

## Introduction

Most postindustrial nations face a fundamental dilemma: while the gender gap in mathematics achievement is closing, females are still deciding against studying and working in science, technology, engineering, and mathematics (STEM; Dasgupta, 2011; Legewie and DiPrete, 2012; Ceci et al., 2014; Hyde, 2014). The percentage of women receiving degrees in STEM languishes around 30%, even though in most Western societies more than half of college degrees are obtained by women (OECD, 2006, 2007; Hill et al., 2010; Friedman-Sokuler and Justman, 2016). The same pattern can be observed in non-academic careers: although women represent about half of the general labor force in Western countries, they occupy only 24% of the positions in STEM working fields (Halpern et al., 2007; OECD, 2007; Friedman-Sokuler and Justman, 2016).

Historically, women’s underrepresentation in scientific fields has been explained by their alleged lower innate ability in mathematics, one key subject of STEM. However, research has challenged this claim (Friedman, 1989; for overviews see Ceci et al., 2014; Hyde, 2014), with evidence showing that the gender gap in factual mathematics achievement has narrowed considerably in the past decades (Hyde et al., 1990a, 2008; Hyde and Mertz, 2009; Else-Quest et al., 2010; Lindberg et al., 2010). Now, differences in self-efficacy between girls and boys are by far the most frequently cited explanation for the gender gap in STEM (Kanny et al., 2014). One particularly relevant variable is self-efficacy beliefs in mathematics, which consistently predicts educational achievement and career outcomes above and beyond prior performance (Pajares, 1996; Richardson et al., 2012; Cheema and Galluzzo, 2013; Galla et al., 2014; Larson et al., 2015). Thus, “performance and self-efficacy don’t always go hand in hand” (Dasgupta, 2011, p. 232). It seems that girls – although they receive similar grades to boys – are more likely to lack confidence in their mathematical abilities (Catsambis, 1994; Tiedemann, 2000; Else-Quest et al., 2013).

To explain this apparent contradiction, it is crucial to examine the reasons for the relatively low self-efficacy beliefs of girls, which are not yet well understood. The current paper draws on data from over 1,000 secondary school students to examine three sources of self-efficacy beliefs in mathematics: mastery, social persuasion in the form of positive feedback and encouragement from others, and exposure to positive vicarious experiences.

The current study extends previous research in two ways. First, we differentiate between three dimensions of mastery. Traditionally, mastery experience has been measured by asking students to recall their mathematics grades. In addition to this, the current study measures two *situational* mastery experiences, assessed immediately after taking a standardized mathematics test. These are state self-esteem (an affective measure reflecting how people felt about their test performance), and self-enhancement (a meta-cognitive measure reflecting the gap between subjective and objective ratings of test performance). In doing so, we are able to nuance between how objective indices of achievement differ from subjective interpretations of one’s achievements in terms of shaping prospective self-efficacy beliefs of girls and boys. Second, we draw on sociometric methods to examine the role of social persuasion and vicarious experiences on self-efficacy. As such, the current study moves beyond the reliance on self-report measures that has caused ambiguity in the conclusions that can be drawn from previous research.

## Theoretical Framework

### Mathematics Self-Efficacy: Outcomes and Predictors

Learners’ confidence in their skills and capabilities to succeed in certain tasks – irrespective of their actual performance – is frequently described as self-efficacy (Pajares, 1996; Bandura, 1997). Self-efficacy beliefs predict how long students will persist at a task in order to solve it (Pajares, 2005; Paunonen and Hong, 2010) and how effortful they engage in academic situations as rated by their teachers (Galla et al., 2014). Moreover, bolstering self-efficacy beliefs has been shown to have a positive effect on self-regulation and performance (Schunk and Ertmer, 2000). Partly for these reasons, higher self-efficacy beliefs among students are associated with higher achievement, above and beyond differences in prior performance (Multon et al., 1991; Parker et al., 2014; Schöber et al., 2018; for a review see Richardson et al., 2012).

In his social cognitive theory, Bandura (e.g., 1997, 2001) distinguished between four sources of self-efficacy: mastery experiences, social persuasion, vicarious experiences, and physiological states (note that the current manuscript focuses on only the first three of these sources; we did not measure physiological states). Mastery experiences, often assessed as grades in the relevant domain, refer to past experiences of success and failure, and are typically considered to be the largest predictor of self-efficacy beliefs (Bandura, 1986, 1997; Britner and Pajares, 2006; Usher and Pajares, 2008; Byars-Winston et al., 2017). In contrast to mastery experiences and physical or affective states, which are intrapersonal sources of self-efficacy, social persuasion and vicarious experiences are interpersonal sources of self-efficacy, grounded in the social environment. Social persuasion is typically conceptualized as realistic, positive feedback from others about one’s abilities. Several studies show an association between social persuasion and the self-efficacy beliefs of students (Joët et al., 2011; Phan, 2012; Lau et al., 2018; for an overview see Usher and Pajares, 2008), although the relationship is often weak when controlling for the other sources (Byars-Winston et al., 2017). The specific source of social persuasion might also be of importance: in some studies, social persuasion by family members and peers was most important (Ahn et al., 2016), while in other studies only social persuasion by teachers predicted self-efficacy (Ahn et al., 2017; Won et al., 2017).

Vicarious experiences refer to the extent that people have examples of good or poor performance in a particular task in their life. Vicarious experiences are thought to affect self-efficacy in the sense that observing outcomes of significant others as models can be experienced as indicative of one’s own capabilities. Bandura (1994) theorized that, while similarity to the model performing a task will be particularly relevant for the model’s effects on self-efficacy beliefs, students will also be likely to seek upward comparisons to models who possess the competencies they aspire to acquire. So far, researchers have not succeeded in demonstrating the exact nature of the association empirically. It remains unclear which type of models (e.g., similar or more competent peers, prestigious adults) exert which kind of influence on students’ self-efficacy (Usher and Pajares, 2008; Joët et al., 2011). In one study, vicarious model experience from teachers was a significant positive predictor of self-efficacy in 2,893 middle school students (Ahn et al., 2016). In other studies, however, neither vicarious experience from teachers nor from family or peers significantly predicted self-efficacy when controlling for the other sources (Ahn et al., 2017; Byars-Winston et al., 2017).

### Mathematics Self-Efficacy and Gender

It has been consistently reported that girls show lower mathematics self-efficacy than boys (OECD, 2012a, 2014a; for a meta-analysis see Huang, 2013), and that this difference partly explains the gender gap in the choice of a career in STEM. Hackett and Betz (1981) were the first to suggest that the gender differences in career-relevant self-efficacy might be due to girls and boys having different access to the sources of self-efficacy. For example, in a study with 3rd-grade elementary school students, Joët et al. (2011) found that girls reported lower levels of mathematics mastery than boys. In a study by Lent et al. (1996) with high school students, however, no difference in mathematics-related accomplishments in terms of gender became apparent.

With respect to interpersonal sources of self-efficacy, evidence has been mixed. Girls in elementary school reported receiving less social persuasion than boys in mathematics, and comparable vicarious experiences (Joët et al., 2011). In contrast, girls in high school report somewhat more persuasive and vicarious experiences than boys (Lent et al., 1996).

The focus of these studies is on identifying overall differences between girls and boys in their access to the different sources of self-efficacy. Other researchers have focused on how the different sources of self-efficacy are weighted differently by girls and boys in terms of the extent to which they predict self-efficacy. For academic self-efficacy beliefs, Usher and Pajares (2006) identified mastery experiences as a strong predictor for both genders, whereas social persuasion was only a strong predictor of self-efficacy for girls. In a qualitative study, Zeldin and Pajares (2000) explored the narratives of women in mathematical, scientific, and technological careers. The authors found that women most frequently mentioned social persuasion and vicarious experiences as sources of their self-efficacy beliefs in these domains. Analyzing the data of 2,511 upper-elementary and middle school students, Butz and Usher (2015) found that mastery experience and social persuasion were the most frequently reported sources of self-efficacy, but girls reported social sources more often than boys. In an analysis of 331 physics students, Sawtelle et al. (2012) found that vicarious learning was most important for predicting physics self-efficacy among women, whereas mastery experience was the strongest predictor among men. In sum, research across these domains suggests that intrapersonal sources (i.e., mastery experiences) might be an especially relevant predictor of boys’ mathematics self-efficacy, whereas interpersonal sources (i.e., social persuasion and vicarious experiences) might be relatively more important for girls’ mathematics self-efficacy.

### Examining “Live” Mastery Experiences: Self-Enhancement and State Self-Esteem After a Test

Traditionally, operationalizations of mastery experiences have used either (self-reported) previous grades from official rosters (Matsui et al., 1990; Klassen, 2004) or items inquiring about grades (e.g., “I got a high grade in last year’s math class”, Lent et al., 1991; “I am capable of receiving good grades on my assignments in this class”, Sawtelle et al., 2012; Dou et al., 2016). These measures are based on school marks or performance in class, representing an external, distal evaluation.

To better understand mastery experiences, we argue that it would be beneficial to tap into the “live” experiences and interpretations of a situation in which abilities are demonstrated, rather than simply measuring previous achievements. How students *feel* about themselves and their performance in such situations and how they *think* they performed can be relevant. To our knowledge, the current study is the first to directly test the relative contribution of these factors to boys’ and girls’ mathematics self-efficacy. We describe these measures in more depth below.

Performance-related state self-esteem (Heatherton and Polivy, 1991) represents an affective interpretation of one’s own performance and can be assessed directly following a test situation. As Heatherton and Polivy (1991) point out “James (1890) described self-esteem as similar to a barometer that rises and falls as a function of one’s aspirations and success experiences. He also noted that there is a certain average tone to the self-feelings people maintain that is largely independent of objective feedback that might contradict the self-concept” (p. 895). In line with this, we argue that performance-related state self-esteem might contribute to our understanding of mastery experience. Further, we expected to find substantial gender differences in state self-esteem, given that girls report higher levels of math anxiety and less positive affect toward mathematics than boys (Betz, 1978; Hyde et al., 1990b; Else-Quest et al., 2010; Nosek and Smyth, 2011). Thus, it seems plausible that girls will also report lower state self-esteem after a mathematics test, which, in turn, could lead them to be less optimistic about their capability to master future challenges.

Self-enhancement, defined as unrealistically positive self-views (Alicke and Sedikides, 2009), represents a meta-cognitive interpretation of one’s performance in a situation. Like state self-esteem, it is well suited to being assessed immediately following an actual test situation. This construct is more specific than academic self-concepts, which have been defined as students’ perception of their competence at a given activity (e.g., Wigfield and Eccles, 2000). In contrast, self-enhancement taps into illusory competence beliefs; that is, the subjective, psychological component of self-confidence that is left once objective performance is taken into account. Self-enhancement is also conceptually distinct from self-efficacy: whereas self-efficacy is directed to anticipated future events, self-enhancement contains interpretations of past or present events. Past research suggests that girls self-enhance less in mathematics than boys (Kurman, 2004). This could be one explanation for girls reporting less mastery experiences in mathematics, even when they obtain equal or better grades. This, in turn, might contribute to their lower self-efficacy beliefs.

### Distal Measures for the Assessment of Interpersonal Sources

In the past decade, several researchers have called for the development of new measures for the interpersonal sources of self-efficacy. This is due to the unsatisfactory reliability of the available items, specifically regarding vicarious experiences (Usher and Pajares, 2008; Ahn et al., 2017). While Ahn et al. (2017) focused on nuancing interpersonal sources by assessing different social models, we propose sociometric data as novel measures of interpersonal sources of self-efficacy. Using distal measures of environmental variables – meaning that the information they contain is not obtained from the individual itself but from its social environment – promises to add explanatory power when predicting outcomes (Fiedler, 2014).

Typically, researchers have evaluated interpersonal sources of self-efficacy by using self-report measures. In order to form a social persuasion score, students in previous research have been asked whether they received encouraging messages about their academic abilities from significant others (e.g., “My classmates said that I understood everything taught in class”, Hampton, 1998; “People often tell me that I am a good mathematics student”, Usher and Pajares, 2006; “When I am struggling with math, my teacher tells me that I can do well”, Ahn et al., 2017). In doing so, it remains unclear whether students actually receive this encouragement, or whether self-reported levels of received social persuasion are biased by one’s own efficacy beliefs (Ahn et al., 2017).

We propose that being asked for advice in a particular domain is a straightforward ascription of one’s competence by others. Thus, in the present research, social persuasion is measured by assessing the number of classmates who – in their own questionnaires – indicate that they would ask the respective peer for advice if they had mathematics-related problems. A meaningful association between the number of nominations a student receives from peers and social persuasion has been shown in recent research (Dou et al., 2016). Furthermore, children aged 7–11 years use environmental cues to infer the existence of peer relationships (Neal et al., 2014), which is particularly straightforward in the case of help and advice seeking. It can therefore be assumed that students who are frequently named by their classmates as popular math helpers are aware of their popularity, and hence, are benefiting in terms of social persuasion.

Similarly, we designed our survey to provide more objective information about vicarious experiences. Vicarious experiences are typically assessed by various forms of self-report in which students are asked to rate their degree of exposure to peer or adult models (e.g., “I have a friend who wants to have a math-related career”, Ahn et al., 2017; “Many of the adults I know have good math skills”, Lent et al., 1991). However, asking students about their comparison partner does not give objective information regarding the performance level and academic standing of that person. In the present research, vicarious experience was therefore assessed by measuring the model’s achievement directly (rather than via self-report by the participant) once the participant indicated the preferred model. To deduce the models’ competence-related status in the respective classroom environment, their achievement was located with respect to the mean achievement of all comparison partners chosen by other classmates (i.e., group mean centering; cf., Aiken and West, 1991). Doing so allowed us to identify whether students with high modeling aspirations (i.e., the ones really choosing more competent models than their classmates, and not just perceiving them as more competent) would show higher levels of self-efficacy.

## The Present Research

With girls and boys showing increasingly similar mathematics performance, the question arises why girls nevertheless keep reporting lower self-efficacy in mathematics. In order to better understand this, the current research investigates gender differences in mathematics self-confidence *following* a test performance.

**FIGURE 1:**
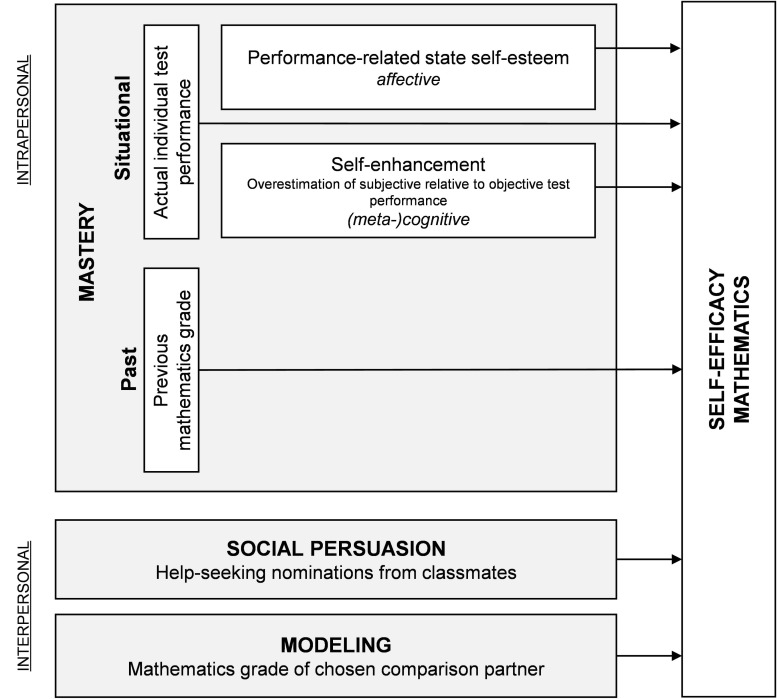
Conceptual model of the predictors of mathematics self-efficacy including (proxy) measures.

Complementing the traditional method of asking participants to retrospectively report previous grades to assess mastery experiences, the present study includes two measures assessed *immediately after* a mathematics test: state self-esteem and cognitive self-enhancement. Furthermore, interpersonal predictors of self-efficacy are assessed by using objective proxies. Students’ popularity as a source of advice in their mathematics classes serves as an indicator of social persuasion. Students’ modeling choices are captured by directly measuring the academic achievement of vicarious models. The constructs measured in the current study are summarized in Figure 1. Physiological and affective states before the test performance were not assessed in this study.

Consistent with previous research, we hypothesized that girls would show lower levels of mathematics self-efficacy than boys (Hypothesis 1). We further predicted that girls would score lower than boys both on self-enhancement (Hypothesis 2a) and state self-esteem (Hypothesis 2b). Given the lack of consistency in previous research – and given that we are introducing unconventional distal measures of the interpersonal sources – we did not formulate direct hypotheses regarding social persuasion and vicarious experiences. However, we expected that all assessed sources should significantly predict mathematics self-efficacy above and beyond achievement in terms of test performance (Hypothesis 3). We further examined whether different sources predicted girls’ and boys’ self-efficacy. On the basis of the research that relied on self-report measures (Sawtelle et al., 2012; Butz and Usher, 2015), we predicted that boys’ self-efficacy beliefs would be more influenced by intrapersonal influences (i.e., self-enhancement and state self-esteem), whereas girls’ self-efficacy would be more influenced by interpersonal influences (i.e., social persuasion and vicarious experiences; Hypothesis 4).

## Method

### Sample

To test our hypotheses, we used a subsample of a dataset collected as part of a larger study on educational adjustment of adolescents. Material on all measures relevant for the present study was administered to 1,007 secondary school students in 48 classrooms (*M* = 20.98, *Min* = 8, *Max* = 32) in Germany. In 10 classrooms, scales for the evaluation of own performance in the test were not administered due to a misunderstanding among research assistants. Of the 813 remaining students, 28 did not nominate a comparison partner and another 20 nominated themselves and were thus excluded from the analyses. For one student, there was missing information regarding his or her gender. Given our interest in gender differences, this student was also excluded from the analyses. The excluded cases (*n* = 243) did not differ from our final sample (*n* = 764) with regard to mathematics self-efficacy (*t* = −0.265, *p* = 0.791), state self-esteem (*t* = −1.419, *p* = 0.156), and the social persuasion score (*t* = −1.092, *p* = 0.275). However, significant differences were found for students’ self-reported mathematics grade (*t* = 4.039, *p* ≤ 0.001), students’ test performance (*t* = −12.236, *p* ≤ 0.001), the mathematics grade of the comparison partner (*t* = 4.552, *p* ≤ 0.001), and the self-enhancement score (*t* = 2.053, *p* ≤ 0.05). In comparison to the excluded cases, the students in our final sample had better grades in mathematics, showed a higher test performance in the standardized mathematics test, chose comparison partners with better grades in mathematics and were less likely to self-enhance. This can be explained by the fact that the classrooms, in which the material relevant to this study was not administered, were all lower track classrooms.

The final dataset thus consisted of 764 9th graders of German secondary schools (56.8% female, *M*_age_ = 15.43, *SD*_age_ = 0.81, *Min*_age_ = 13 years, *Max*_age_ = 18 years). Of this sample, 42.1% reported that at least one of their parents were born in another country than Germany. Regarding the different secondary school types, 80.0% of the students attended the *Gymnasium* (higher school track), 12.2% the *Realschule*, and 7.9% the *Hauptschule* (lower school tracks). In order to assess socioeconomic background, students indicated the approximate number of books in their home. This measure has repeatedly been applied in international assessments of educational attainment (e.g., OECD, 2012b, OECD, 2014b, OECD, 2017a) and shown to be an adequate proxy for the educational, social, and economic background of the students’ families (e.g., Ehmke and Siegle, 2005; Watermann and Baumert, 2006). Of the 720 students who provided information on the number of books in their home, 0.3% reported “none”, 4.3% “1–10 books”, 12.1% “11–50 books”, 17.2% “51–100 books”, 22.5% “101–250 books”, 23.5% “251–500 books”, and 20.1% “more than 500 books”. To obtain more precise estimates, reliability analyses as well as grand and group means for the classrooms were obtained from the larger dataset of 1,007 students.

### Procedure

With the consent of schools, teachers, and parents, questionnaires were administered during regular class hours. Students were told that we were interested in how they see themselves, and what they think and feel. They were informed that participation was voluntary, reassured that there were no right or wrong answers, and encouraged to respond as spontaneously as possible in whatever way seemed right for them. Parents were given the option to withdraw their consent even after their children participated in the survey. Anonymity of the data collection and processing was explained and granted.

First, students were given 15 min to work on a standardized performance test in mathematics. They were then asked to estimate the percentage of items that they had answered correctly before reporting their state self-esteem. As a next step, students were asked to provide information on their social networks within the classroom (procedure see below) and to answer the items assessing mathematics self-efficacy. Grades on the previous school report were assessed via self-report at the end of the questionnaire, together with socio-demographic information. All participants who had answered the questionnaire with ostensible diligence took part in a lottery drawing of goods (e.g., books) or vouchers.

### Measures

#### Mathematics Self-Efficacy

To measure the outcome variable of our main analyses, we used a well-established German measure of academic self-efficacy (Jerusalem and Satow, 1999). This measure was adapted for the subject of mathematics by asking students to think about the subject mathematics when answering the items. Example items from the 7-item measure include: “If I try hard enough, I can even solve difficult assignments”, “I am sure I can perform well even if the teacher is doubting my abilities”, and “Even if I would be sick for a longer period of time, I would perform well.” Students indicated their agreement on a 5-point Likert scale (1 = *not at all true*, 5 = *exactly true*). There was one negatively phrased item, which was removed from the scale due to a notably increased internal consistency (from α = 0.82 to α = 0.89).

#### Past Mastery Experience

In line with traditional operationalizations of mastery experience, we assessed students’ self-reported mathematics grade they had obtained on their last report card (Matsui et al., 1990; Klassen, 2004). Grades were recoded so that higher values indicated higher achievement.

#### Situational Mastery Experience: Individual Test Performance

Students’ test performance was assessed using items from the advanced mathematics test of the Third International Mathematics and Science Study (TIMSS). The test comprised the three content areas of numbers, algebra, and geometry, and has been repeatedly and successfully applied in international student assessments (e.g., Baumert et al., 1999). The test assesses the extent to which students understand and utilize conceptional thinking, problem solving, and application. TIMSS has been designed with extensive input from experts in mathematics and science education, assessment, and curriculum within each participating country (e.g., Burns et al., 2011). Although TIMSS has – especially across states in the United States – been found to be not particularly curriculum-sensitive (Schmidt et al., 1998, 2005), about 80–95% of the mathematical items – depending on the age group – can be classified as “curriculum valid” for Germany (Baumert et al., 1998; Wendt et al., 2016). Before conducting our study, 30 items were pretested and 10 items showing extreme means, and zero or nearly zero variances were eliminated. The 20 items we used in the main study were selected so that (a) the proportion of items with medium difficulty was largest and (b) the item difficulty was between 0.20 and 0.80. Reliability was calculated across the different content categories and was determined to be satisfactory (α = 0.75).

#### Situational Mastery Experience: Cognitive Component

Self-rated performance was assessed by asking students to indicate how many items they have answered correctly on a 60mm horizontal line. A mark at the far left indicated a very low performance evaluation (*none correct*) and a mark at the far right indicated a very high performance evaluation (*all correct*). Distances from the left end to the mark were sized and transformed into percentages (*M* = 66.60, *SD* = 20.73, *Min* = 0%, *Max* = 100%). To obtain the self-enhancement index, we regressed self-reported performance on the actual performance score and saved standardized residuals (John and Robins, 1994; see also Dufner et al., 2015).

#### Situational Mastery Experience: Affective Component

Students’ affective interpretation of their performance situation in the mathematics test was assessed by participants’ state self-esteem directly after the performance. Students rated their state self-esteem using an adapted version of the 7-item subscale of Heatherton and Polivy’s (1991) State Self-Esteem Scale (SSES; e.g., “I feel frustrated or rattled about my performance”, “I feel confident that I understand things”). All items used a 5-point Likert response scale (1 = *not at all true*, 5 = *exactly true*) and formed a reliable scale (α = 0.81).

#### Social Persuasion

Students were asked to provide a maximum number of three classmates whom they would ask for help regarding learning and homework in mathematics. We were not interested in the number of outgoing nominations but rather the number of incoming nominations a student received from his or her classmates. We argue that this measure, referred to in social networks as the *Indegree* (cf., Hanneman and Riddle, 2011), reflects a distal proxy of ascribed competence by other students in the classroom. To obtain the social persuasion score, each participant’s Indegree in each classroom’s advice network was calculated and normalized by class size and thus, by the maximum number of incoming nominations from classmates using UCINET (Borgatti et al., 2002), such that the absolute number of incoming nominations was divided by the maximum possible Indegrees to allow for comparison between classroom networks with different sizes (Hanneman and Riddle, 2011). Due to the high skewness of the social persuasion score, the logarithm ln(score+1) was taken for all analyses.

#### Vicarious Experiences

Bandura proposed that vicarious experiences affect efficacy beliefs in that comparison to the outcomes of significant others could be experienced as indicative of one’s own capabilities (Bandura, 1994). In order to capture this, before taking the test, students were asked to indicate one classmate whom they would like to compare the results of their mathematics test with once they were finished. To obtain a proxy for vicarious experiences, we retrieved the mathematics grade of the nominated classmate from the data set. We regarded the grade of the respective classmate as a more relevant proxy than the classmate’s test performance given that students in a classroom are more likely to be informed about the grade of a friend, whereas the performance in the test just taken was not yet known. Bandura further theorized that, while comparison to similar others performing a task could be particularly relevant to self-efficacy beliefs, students will also be likely to seek out models with status and prestige. To deduce the model’s competence-related status in the respective classroom environment, his or her achievement was group mean centered (cf., Aiken and West, 1991), subtracting the mean achievement of all comparison partners chosen by other classmates. Doing so allowed us to identify whether students who chose more competent vicarious models than the average classmate would show higher levels of self-efficacy than students who chose less competent models than the average classmate. The majority of students in our sample indicated that they were friends with the chosen comparison partner. Altogether, only 46 out of the 764 students (6.0%) indicated that they were not friends with their chosen comparison partner. Eight of the students who chose a valid comparison partner (1.0%) did not indicate whether they were friends with the comparison partner or not. With regard to same- and opposite-sex choices, only 42 male students chose a female comparison partner (13.0%) and only 51 female students chose a male one (11.8%).

### Data Analytic Strategy

At first, measurement invariance across gender was tested for our multi-item measures mathematics self-efficacy and state self-esteem in order to determine the degree of consistent measurement across groups. Doing so allowed us to test whether both measures were comparable in our subsamples of boys and girls, constituting a prerequisite for meaningful group mean comparisons.

In a second step, we estimated descriptive statistics and bivariate correlations for all variables of interest. In addition, we tested mean differences and standardized mean differences between girls and boys using linear regression analyses with a dummy variable taking a value of zero for boys and one for girls.

Next, we regressed students’ mathematics self-efficacy on their test performance using a multilevel multiple-group linear regression model, stratified by gender. To test whether regression parameters in the overall model differed significantly for girls and boys, we compared an unconstrained model allowing for varying parameters between girls and boys to a constrained model in which regression parameters were set equal using a chi-square difference test.

Subsequently, we regressed mathematics self-efficacy on our additional predictor variables: students’ grade in mathematics, students’ self-enhancement score, students’ state self-esteem after the performance test, the social persuasion score (logarithmized), and the grade in mathematics of the chosen comparison partner, while accounting for students’ actual mathematics achievement (i.e., performance in the administered test). Again, a multilevel multiple-group regression analysis, stratified by gender, was conducted and differences in regression parameters between girls and boys were tested using a chi-square difference test.

Finally, we conducted mediation analyses to examine whether substantial parts of gender differences in self-efficacy can be traced back to gender differences in our predictor variables – above and beyond test performance.

Unless stated differently, analyses were run with Mplus version 8.1 (Muthén and Muthén, 1998–2012) using a robust maximum likelihood estimator. The multilevel regression models for girls and boys were estimated simultaneously using the GROUPING command within Mplus (0 = boys, 1 = girls). The complex structure of the data (students nested within classrooms), which may violate the assumption of independent observations within regression analyses (e.g., Snijders and Bosker, 2012), was accounted for by using the TYPE = COMPLEX command within Mplus. Missing data were treated following a full information maximum likelihood approach, which has been shown to yield unbiased parameter estimates and to retain high statistical power (Schafer and Graham, 2002; Enders, 2010). In all analyses, the type of school (dummy coded: 0 = lower academic track; 1 = upper academic track), students’ general academic self-efficacy, and students’ subjective importance of being good in mathematics were used as missing data correlates/auxiliary variables (Muthén and Muthén, 1998–2012). In order to avoid listwise deletion of individuals with missing data on x-variables, independent variables were treated as dependent variables within Mplus (Hox et al., 2015). All variables except for the self-enhancement score were group mean centered. Accordingly, slopes are interpreted as the increase in the criterion variable associated with one unit increase in the predictor variable – relative to the classroom’s mean.

## Results

### Preliminary Analyses

Before conducting our main analyses, we tested measurement invariance across gender for our two multi-item measures mathematics self-efficacy and state self-esteem within the framework of multiple-group confirmatory factor analyses. For both measures, scalar invariance was supported and thus, the statistical prerequisites for mean value comparisons between both groups were met. For the detailed analyses see the concomitant supplement (Supplementary Analyses).

### Descriptive Analyses

In Table 1, descriptive statistics for our dependent and independent variables are shown. Mean values and standard deviations are presented for the total sample as well as separately for girls and boys. As can be seen, there were no gender differences in previous mathematics grades (*B* = −0.093, *p* = 0.300, *d* = 0.093), although boys did outperform girls in the standardized mathematics test we administered (*B* = −4.941, *p* ≤ 0.001, *d* = 0.392). In line with Hypothesis 1, girls reported significantly lower self-efficacy in mathematics than boys (*B* = −0.405, *p* ≤ 0.001, *d* = 0.472). Moreover, girls showed significant lower levels of self-enhancement (*B* = −0.437, *p* ≤ 0.001, *d* = 0.446) and reported significantly lower levels of performance-related state self-esteem than did boys (*B* = −0.375, *p* ≤ 0.001, *d* = 0.443), thereby confirming Hypotheses 2a and 2b. No gender differences were found, however, for students’ social persuasion scores (*B* = 0.034, *p* = 0.751, *d* = 0.025) or for the grades of classmates chosen as comparison partners (*B* = −0.157, *p* = 0.081, *d* = 0.157).

**TABLE 1:** Summary of means and standard deviations as a function of gender.

		**Mathematics self-efficacy**	**Test performance^*a*^**	**Mathematics grade^*a*^**	**Self-enhancement score (cognitive)**	**State self-esteem (affective)^*a*^**	**Social persuasion score^*b*^**	**Mathematics grade of CP^*a*^**
								
	***N***	***M* (*SD*)**	***M* (*SD*)**	***M* (*SD*)**	***M* (*SD*)**	***M* (*SD*)**	***M* (*SD*)**	***M* (*SD*)**
Total sample	764	3.57 (0.88)	0.15 (12.84)	0.01 (1.00)	−0.02 (1.00)	−0.01 (0.87)	1.58 (1.35)	0.01 (1.01)
Girls	434	3.40 (0.87)	−1.98 (12.69)	−0.03 (0.99)	−0.21 (0.99)	−0.17 (0.87)	1.60 (1.31)	−0.06 (0.98)
Boys	330	3.80 (0.83)	2.96 (12.50)	0.06 (1.02)	0.23 (0.97)	0.20 (0.82)	1.56 (1.41)	0.09 (1.04)
*B* (*SE*)		−0.405 (0.07)	−4.941 (1.06)	−0.093 (0.09)	−0.437 (0.07)	−0.375 (0.06)	0.034 (0.11)	−0.157 (0.09)
Sig. (2-tailed)		0.000***	0.000***	0.300	0.000***	0.000***	0.751	0.081

Numbers in the bottom two rows represent the results of simple regression analyses with gender as the independent variable. All values were estimated using Mplus and full information maximum likelihood. Standard errors were adjusted for the nesting of students within classes. Gender: 0 = boys, 1 = girls. CP = comparison partner. ^ a^ This variable was centered around the group mean. ^*b*^ Due to the high skewness of the social persuasion score, ln(score+1) was taken for all analyses. ***p ≤ 0.001.

Table 2 summarizes the intercorrelations among our measures. Significant positive correlations emerged between students’ mathematics self-efficacy and the presumed sources of self-efficacy. Because there were moderate to strong correlations between some of our predictor variables, we tested multicollinearity by means of the variance inflation factor (VIF) associated with each independent variable. We examined VIFs using SPSS (version 25.0; IBM Corp, 2017) based on a multiple regression analysis of students’ self-efficacy on all predictor variables. With the lowest VIF-score being 1.083 and the highest being 1.757, no multicollinearity was indicated.

**TABLE 2:** Correlations of the dependent and independent variables.

		**1**	**2**	**3**	**4**	**5**	**6**	**7**	**8**	**VIF**
1	Mathematics self-efficacy	1								
2	Test performance^*a*^	0.257***	1							1.328
3	Mathematics grade^*a*^	0.485***	0.351***	1						1.757
4	Self-enhancement score (cognitive)	0.283***	0.008	0.152***	1					1.265
5	State self-esteem (affective)^*a*^	0.433***	0.308***	0.350***	0.417***	1				1.493
6	Social persuasion score^*b*^	0.307***	0.261***	0.546***	0.075*	0.189***	1			1.532
7	Mathematics grade of CP^*a*^	0.159***	0.140***	0.242***	0.014	0.102**	0.157***	1		1.083
8	Gender	−0.217***	−0.191***	–0.043	−0.208***	−0.214***	0.013	–0.076	1	1.129

*N = 764. Correlations were estimated using Mplus and full information maximum likelihood. Standard errors were adjusted for the nesting of students within classes. VIF = Variance inflation factor of all predictor variables (variables 2–8; results were estimated using SPSS). Gender: 0 = boys, 1 = girls. CP = comparison partner. ^*a*^ This variable was centered on the group mean. ^*b*^ Due to the high skewness of the social persuasion score, ln(score+1) was taken for all analyses. ^∗^*p* ≤ 0.05, ^∗∗^*p* ≤ 0.01, and ^∗∗∗^*p* ≤ 0.001.*

### Multilevel Multiple-Group Regressions

In the next step, we conducted a set of multilevel multiple-group linear regression models. As shown in Table 3 (Model 1), test performance was a positive and significant predictor of self-efficacy for both girls (β = 0.211, *p* ≤ 0.001) and boys (β = 0.232, *p* ≤ 0.001). Subsequently, we tested whether students’ grade in mathematics, self-enhancement score, state self-esteem after the performance test, social persuasion score, and the comparison partner’s mathematics grade are relevant predictors of mathematics self-efficacy above and beyond test performance (Hypothesis 3). We also tested whether different sources are relevant for the formation of mathematics self-efficacy in girls as compared to boys (Hypothesis 4).

**TABLE 3:** Regression models predicting students’ mathematics self-efficacy for girls and boys.

	***B* (*SE*)**				**β**		
	**Girls**		**Boys**		**Girls**	**Boys**	**χ^2^ (*df*)**
**Model 1**							
Test performance^*a*^	0.015	(0.004)***	0.016	(0.004)***	0.211	0.232	0.038 (1)
**Model 2**							
Test performance^*a*^	–0.001	(0.003)	0.005	(0.004)	–0.011	0.069	
Mathematics grade^*a*^	0.300	(0.053)***	0.295	(0.064)***	0.336	0.356	
Self-enhancement score (cognitive)	0.036	(0.050)	0.174	(0.047)***	0.041	0.202	
State self-esteem (affective)^*a*^	0.311	(0.068)***	0.115	(0.069)	0.307	0.112	
Social persuasion score^*b*^	0.057	(0.042)	0.023	(0.041)	0.084	0.038	
Mathematics grade CP^*a*^	0.012	(0.035)	0.050	(0.036)	0.013	0.062	7.704 (6)

*N = 764. All values were estimated using Mplus and full information maximum likelihood. Standard errors were adjusted for the nesting of students within classes. In the last column, χ^2^ differences between a fully unconstrained model and a model assuming equal regression parameters between girls and boys are indicated. CP = comparison partner. ^*a*^ This variable was centered on the group mean. ^*b*^ Due to the high skewness of the social persuasion score, ln(score+1) was taken for all analyses. ^∗∗∗^*p* ≤ 0.001.*

As shown in Table 3 (Model 2), mathematics grade was predictive of both girls’ (β = 0.336, *p* ≤ 0.001) and boys’ (β = 0.356, *p* ≤ 0.001) mathematics self-efficacy. As expected, students who had demonstrated mastery of mathematics in their most recent course reported higher self-efficacy.

For boys, self-enhancement immediately after the test also predicted self-efficacy – over and above the other predictors (β = 0.202, *p* ≤ 0.001). Thus, the more they overestimated their performance on the test, the higher they rated their capability to successfully handle future challenges in mathematics (i.e., self-efficacy in mathematics).

For girls, self-enhancement was not a relevant predictor of self-efficacy in mathematics, but state self-esteem was (β = 0.307, *p* ≤ 0.001). The better they felt about themselves immediately after taking the mathematics test, the higher they rated their self-efficacy for future challenges in mathematics. Another way of interpreting this effect is that the more negatively girls felt about themselves immediately after taking the test, the lower they rated their capability to deal with future challenges in mathematics.

Interestingly, although social persuasion and the comparison partner’s mathematics grade were positively correlated with self-efficacy overall, they did not feature as significant unique predictors when controlling for the other variables (social persuasion: β_*g*__irls_ = 0.084, *p* = 0.178, β_*b*__oys_ = 0.038, *p* = 0.584; mathematics grade of comparison partner: β_*g*__irls_ = 0.013, *p* = 0.741, β_*b*__oys_ = 0.062, *p* = 0.159). The overall model explained a total of 34.4% of the variance in mathematics self-efficacy for girls and a total of 29.8% for boys. Cohen’s *f*^2^ statistic yielded effect size estimates of 0.52 for girls and 0.42 for boys, which are considered large effects (Cohen, 1988).

In addition to examining each predictor’s significance, we examined whether the regression weights differed significantly for girls and boys. Hereby, we compared two sets of regressions: one that allowed for varying parameters between girls and boys, and one which artificially constrained the model such that regression parameters between girls and boys were set equal. Chi-square statistics were not significant, χ^2^ (6) = 7.704, *p* = 0.261, which indicates no difference in parameter estimates between girls and boys. Therefore, according to the results of the chi-square difference test, assessed sources were equally important predictors of girls’ and boys’ self-efficacy. This implies that the findings reported above, although significant, need to be interpreted with caution and warrant replication. All models are reported in detail in the supplement (Supplementary Table 1).

### Mediation Analyses

In a final step, we analyzed the direct and indirect effects in mediation analyses (Supplementary Table 2) to examine whether substantial parts of gender differences in self-efficacy can be traced back to gender differences in the predictors – above and beyond their test performance. Here, we found that, in addition to a persisting direct effect of gender on mathematics self-efficacy, there were significant indirect effects through actual achievement in the test (*B* = −0.044, *p* ≤ 0.01), self-enhancement (*B* = −0.047, *p* ≤ 0.01), and state self-esteem (*B* = −0.123, *p* ≤ 0.001).

## Discussion

Consistent with previous research (Pajares, 2005; OECD, 2012a, OECD, 2014a; Huang, 2013) and confirming Hypothesis 1, our data showed that girls have much lower mathematics self-efficacy than boys, even though their math grades did not significantly differ. This study was designed to unpack why this might be the case, using three sources postulated in Bandura’s self-efficacy model. We complemented previous research in two ways. First, we nuanced between three types of mastery. In addition to measuring previous grades (which has traditionally been used as a proxy for mastery experiences), we included an affective and a cognitive component of situational mastery experience. Second, we applied sociometric methods to examine the role of interpersonal factors in contributing to self-efficacy without exclusively relying on the target students’ self-report.

### The Role of Experiences Following a Test Situation

Girls in our sample felt worse about themselves after the mathematics performance test than did boys, and were less likely to overestimate their performance on the task relative to boys. Furthermore, these differences appeared to be consequential: the cognitive component of mastery (i.e., self-enhancement) was a significant predictor of boys’ (relatively high) self-efficacy, while the affective component (i.e., state self-esteem after the test) was a significant predictor of girls’ (relatively low) self-efficacy. Taken together, our findings suggest that including additional dimensions of mastery indeed contributes to the understanding of mastery experiences as a source of self-efficacy. It should be noted that the difference in sources of self-esteem for boys and girls was subtle, and formal moderation analyses were non-significant. Yet, our findings suggest that different cognitive and affective experiences of girls and boys following test situations can potentially contribute to their prospective self-efficacy beliefs.

These findings corroborate previous results of consistently large gender differences in mastery experience in mathematics (Lent et al., 1996; Kurman, 2004; Joët et al., 2011; for an overview see Usher and Pajares, 2008). However, by complementing self-reported grades using a two-pronged approach to measure experiences following test situations, our results go beyond prior findings. Assessing mastery experience solely by inquiring students’ grades or self-reports of achievements cannot provide an unambiguous answer to the question of why girls report lower mastery experience despite obtaining equal grades in mathematics. A crucial factor might be girls’ negative subjective interpretations following performance situations (Pajares et al., 2007; Usher and Pajares, 2008).

Bandura (2001) notes that “people are sentient, purposeful beings. (…) if they construe their failures as presenting surmountable challenges they redouble their efforts, but they drive themselves to despondency if they read their failures as indicants of personal deficiencies” (Bandura, 2001, p. 6). Consistent with this argument, our fine-grained analysis of mastery experiences suggests that the same performance has quite different implications for girls and boys. On an affective level, girls report lower state self-esteem after taking the test in mathematics. This suggests that they not only discount their performance but also let it undermine the way they feel about themselves: besides thinking of tests as failures more often than boys, girls also feel less worthy after taking them – even before its actual outcomes are known. In this vein, our results complement findings by Crocker et al. (2003), who, examining a sample of 122 female and male students majoring in engineering and psychology, found that self-esteem decreased on days they received poor grades. We find that, on a cognitive level, a self-enhancing student appears to experience low performances as less daunting and high performances as more motivating than a student who self-enhances less (Robins and Beer, 2001; Kurman, 2004). Our finding that boys self-enhance more than girls following a mathematics test suggests a potential explanation for the optimistic beliefs they hold about their future performances. The pattern of results in the multilevel multiple-group regressions and the accompanying chi-square tests suggests that future studies need to clarify whether these different levels of state-self esteem and self-enhancement are distinct predictors for boys and girls, respectively, or whether they are equally important for boys and girls.

The results of complementary mediation analyses further show that gender differences on the evaluative dimensions of mastery could account for substantial parts of gender differences in self-efficacy above and beyond actual achievement. It seems that girls’ subjective evaluation of their performance is just as important for inferring capability in future situations as is their objective achievement. Stereotypical expectations, such as that mathematics is “for boys”, translate into stereotype-consistent and performance-depleting self-perceptions (Nosek and Smyth, 2011). Negative stereotypes about girls in mathematics have been found to be internalized by students even before test performance differences emerge (Dasgupta and Stout, 2014). Likewise, parents are less likely to expect their daughters to work in STEM-related careers, regardless of their academic achievement (OECD, 2017b), reflecting gender-specific career stereotypes in students’ environment. Detrimental effects of internalized stereotypes on performance and confidence have been frequently shown for girls and women in the field of mathematics (Steele, 1997; Spencer et al., 1999; Keller, 2002) and could be one cause of the reported results.

### The Role of Interpersonal Factors

A second contribution of the current study is that we drew on actual comparison choices of students in a test situation and sociometric techniques to provide novel indices of these interpersonal sources of efficacy. Using these distal sources instead of self-report data circumvents the possibility that associations between these variables and self-efficacy are merely due to common method variance (cf., Podsakoff et al., 2003). Because our students indicated their model before the test situation (vicarious models) and were nominated as competent helpers by other students (social persuasion), we can infer that our criterion does not overlap with the subjective experience following the test situation. Social psychologists have argued that analytical models contribute more meaningfully to theorizing when intrapsychic processes are linked to extrapsychic (i.e., distal) sources of information than when constructs are measured in similar ways (i.e., both intrapsychic; see Fiedler, 2014).

Although we succeeded in providing new measures of interpersonal sources which – although distal – significantly predicted self-efficacy beliefs, these variables were less relevant in predicting self-efficacy overall and gender differences. Results showed that girls and boys chose equally well-performing models (in terms of grades) and were similarly valued by their peers as competent helpers in the domain of mathematics (vicarious experience and social persuasion, respectively). Furthermore, although these variables were correlated with self-efficacy in the predicted ways, in contrast to our predictions, they ceased to predict significant amounts of variance when other variables were taken into account – for both girls and boys. One of the reasons for this could lie in the distinctive features of our distal measures compared to conventional assessments of interpersonal sources. While self-reported (i.e., perceived) social persuasion might indeed contribute more to girls’ self-efficacy than to boys’, this does not have to be true for social persuasion in the form of *potential* help-seeking nominations from peers (students were asked whom they would ask for help in mathematics in case they encountered difficulties with homework). *Actual* help requests from peers in mathematics could be a stronger proxy and more predictive of self-efficacy. Alternatively, students may not be aware of the potential nominations of their classmates. Future studies could include students’ meta-perceptions of themselves as a source of academic help for their peers (cf., Zander et al., 2018). Combining proximal and distal measures of social persuasion could facilitate our understanding of whether it is more important to girls’ self-efficacy beliefs how much persuasion they *think* they receive versus how much persuasion they *actually* receive from their peers. Analogously, our measure of vicarious models may not be an adequate assessment of actual modeling taking place. Possibly, other classmates rather than the chosen comparison partners are more relevant models. Our results are consistent with previous studies that also frequently failed to identify vicarious models as a source of self-efficacy (see Usher and Pajares, 2008; Joët et al., 2011). Thus, future research needs to continue the effort to develop new measures assessing vicarious experience and exposure to significant models.

### Practical Implications

Bearing in mind that girls and boys differ mostly on the evaluative dimensions of mastery, teachers could help female students forming positive interpretations following test performance. Immediate feedback on their performance could be used to correct girls’ low expectations. Our research shows that girls feel worse and self-enhance less than boys. We find these differences in a situation where students are not aware of their actual performance. In traditional classrooms, it typically takes a few days until students receive feedback about their actual performance from their teachers (often in the form of grades). Since contingency is an important criterion for learning from feedback, we could infer that immediate criterion-oriented feedback following test performances (e.g., by means of digital testing formats) could be an effective tool to improve students’ meta-cognitive skills and narrow the confidence gap between girls and boys. Specifically, girls would immediately learn that they didn’t perform as badly as they felt they did. This could align boys’ and girls’ self-efficacy beliefs.

Likewise, teachers could facilitate female students’ performance-related self-esteem by making them interpret challenges not as a threat to their self-esteem, but as an opportunity to increase their abilities (Dweck, 2000; Yeager and Dweck, 2012). Crocker et al. (2003) found that relying on academic achievement to establish self-esteem costs students more than it supports them: the positive impact of good grades seems to be less influential than the negative impact of bad grades. Instead, encouraging female students to adjust their evaluative response to performance might help to further reduce the gender gap in mathematics self-efficacy. Findings of Kurman (2004) indirectly support this assumption: in a sample of 259 Israeli junior high school students, she found that girls’ self-enhancement in mathematics was positively associated with self-regulated learning behaviors in math 3 months later.

### Limitations and Future Directions

Some limitations of the current study suggest fruitful avenues for future research. First, we conducted our study in the national context of Germany. While Germany is one of the countries where the mathematics gender gap in standardized performance is largest (OECD, 2016), applying the present approach across nations – and possibly relating it to attributes of the assessed countries – might provide further insights on which environmental factors determine the different levels of self-efficacy in girls and boys.

While we think that the use of distal measures is a promising avenue for assessing social persuasion and modeling, these measures should be complemented by conventional self-report scales. Further, our measures of interpersonal sources of self-efficacy are limited in that we solely used sociometric data of peers. Given that peers are particularly influential to the academic and intellectual outcomes of students in adolescence (Wentzel, 2017; Zander et al., 2017; Wang et al., 2018), it is plausible that classmates are a central source of social persuasion. However, we acknowledge that parents and teachers might also be important sources of social persuasion information.

A third limitation concerns the correlational nature of the data, which does not allow causal interpretations regarding self-efficacy and its sources. Data on the sources of self-efficacy were nevertheless assessed before the rating of students’ self-efficacy, or based on reports of other students. As such, it seems plausible that the level of self-efficacy is indeed a consequence of the other assessed experiences, but longitudinal designs could reduce this shortcoming in future research.

Finally, Bandura argued that physiological and affective states provide information about arousal during situations in which the capability in the domain in question is demonstrated. Specifically, Bandura proposes that in stressful situations, people take this proprioceptive information as an indicator of (in)capability, which affects their self-efficacy beliefs (Bandura, 1997). In research that examines sources of self-efficacy, typical assessments of physiological states are measures of anxiety or liking, for example whether thinking of a subject makes them feel sick or depressed (Usher and Pajares, 2008). Thus, a fourth limitation of our study is that physiological states were not assessed. Future research could examine the interrelations between students’ physiological states assessed before or during the test, and students’ affective self-evaluation following the test.

## Conclusion

The present results shed further light on understanding why girls continue to be less successful in standardized large-scale assessments as well as less represented in academic and professional STEM careers than boys. Mathematics self-efficacy has been demonstrated to explain a considerable amount of the gender gap in STEM; increasing it therefore provides one approach to promoting female students. A better understanding of how these differences emerge reveals starting points for teachers to counteract the ongoing disadvantages of girls in mathematics.

## Data Availability Statement

The raw data supporting the conclusions of this manuscript will be made available by the authors, without undue reservation.

## Ethics Statement

Ethical review and approval was not required for the study on human participants in accordance with the local legislation and institutional requirements. Written informed consent to participate in this study was provided by the participants’ legal guardian/next of kin.

## Author Contributions

LZ provided the initial idea, designed the study, organized data collection, performed the first statistical analyses and wrote the first draft of the manuscript. EH and MP performed the statistical analyses presented in the final manuscript and interpreted the results. All authors contributed to the revision of the manuscript and approved the final manuscript.

## Conflict of Interest

The authors declare that the research was conducted in the absence of any commercial or financial relationships that could be construed as a potential conflict of interest.

## References

[B1] Ahn H. S. Bong M. Kim S.-I. (2017). Social models in the cognitive appraisal of self-efficacy information. *Contemp. Educ. Psychol.* 48 149–166. 10.1016/j.cedpsych.2016.08.002

[B2] Ahn H. S. Usher E. L. Butz A. Bong M. (2016). Cultural differences in the understanding of modelling and feedback as sources of self-efficacy information. *Br. J. Educ. Psychol.* 86 112–136. 10.1111/bjep.12093 26387485

[B3] Aiken L. S. West S. G. (1991). *Multiple Regression: Testing and Interpreting Interactions.* Newbury Park, CA: Sage.

[B4] Alicke M. D. Sedikides C. (2009). Self-enhancement and self-protection: what they are and what they do. *Eur. Rev. Soc. Psychol.* 20 1–48. 10.1080/10463280802613866

[B5] Bandura A. (1986). The explanatory and predictive scope of self-efficacy theory. *J. Soc. Clin. Psychol.* 4 359–373. 10.1521/jscp.1986.4.3.359

[B6] Bandura A. (1994). “Self-efficacy,” in *Encyclopedia of Human Behavior*, Vol. 4 ed. Ramachaudran V. S. (New York, NY: Academic Press), 71–81.

[B7] Bandura A. (1997). *Self-Efficacy: The Exercise of Control.* New York, NY: Freeman.

[B8] Bandura A. (2001). Social cognitive theory: an agentic perspective. *Annu. Rev. Psychol.* 52 1–26. 10.1146/annurev.psych.52.1.1 11148297

[B9] Baumert J. Bos W. Klieme E. Lehmann R. Lehrke M. Hosenfeld I. (eds) (1999). *Testaufgaben zu TIMSS/III Mathematisch-Naturwissenschaftliche Grundbildung und voruniversitäre Mathematik und Physik der Abschlußklassen der Sekundarstufe II (Population 3).* Berlin: Max-Planck-Institut für Bildungsforschung.

[B10] Baumert J. Lehmann R. Lehrke M. Clausen M. Hosenfeld I. Neubrand J. (eds). (1998). *Testaufgaben Mathematik TIMSS 7/8. Klasse (Population 2).* Berlin: Max-Planck-Institut für Bildungsforschung.

[B11] Betz N. E. (1978). Prevalence, distribution, and correlates of math anxiety in college students. *J. Couns. Psychol.* 25 441–448. 10.1037/0022-0167.25.5.441

[B12] Borgatti S. P. Everett M. G. Freeman L. C. (2002). *Ucinet for Windows: Software for Social Network Analysis.* Harvard, MA: Analytic Technologies.

[B13] Britner S. L. Pajares F. (2006). Sources of science self-efficacy beliefs of middle school students. *J. Res. Sci. Teach.* 43 485–499. 10.1002/tea.20131

[B14] Burns S. Wang X. Henning A. (eds). (2011). *NCES Handbook of Survey Methods.* Washington DC: U.S. Government Printing Office.

[B15] Butz A. R. Usher E. L. (2015). Salient sources of early adolescents’ self-efficacy in two domains. *Contemp. Educ. Psychol.* 42 49–61. 10.1016/j.cedpsych.2015.04.001

[B16] Byars-Winston A. Diestelmann J. Savoy J. N. Hoyt W. T. (2017). Unique effects and moderators of effects of sources on self-efficacy: a model-based meta-analysis. *J. Couns. Psychol.* 64 645–658. 10.1037/cou0000219 29154576

[B17] Catsambis S. (1994). The path to math: gender and racial-ethnic differences in mathematics participation from middle school to high school. *Sociol. Educ.* 67 199–215. 10.2307/2112791

[B18] Ceci S. J. Ginther D. K. Kahn S. Williams W. M. (2014). Women in academic science: a changing landscape. *Psychol. Sci. Public Interest* 15 75–141. 10.1177/1529100614541236 26172066

[B19] Cheema J. R. Galluzzo G. (2013). Analyzing the gender gap in math achievement: evidence from a large-scale US sample. *Res. Educ.* 90 98–112. 10.7227/RIE.90.1.7

[B20] Cohen J. (1988). *Statistical Power Analysis for the Behavioral Sciences*, 2nd Edn. Hillsdale, NJ: Lawrence Erlbaum Associated, Inc.

[B21] Crocker J. Karpinski A. Quinn D. M. Chase S. K. (2003). When grades determine self-worth: consequences of contingent self-worth for male and female engineering and psychology majors. *J. Pers. Soc. Psychol.* 85 507–516. 10.1037/0022-3514.85.3.507 14498786

[B22] Dasgupta N. (2011). Ingroup experts and peers as social vaccines who inoculate the self-concept: the stereotype inoculation model. *Psychol. Inq.* 22 231–246. 10.1080/1047840X.2011.607313

[B23] Dasgupta N. Stout J. G. (2014). Girls and women in science, technology engineering, and mathematics: STEMing the tide and broadening participation in STEM careers. *Policy Insights Behav. Brain Sci.* 1 21–29. 10.1177/2372732214549471

[B24] Dou R. Brewe E. Zwolak J. P. Potvin G. Williams E. A. Kramer L. H. (2016). Beyond performance metrics: examining a decrease in students’ physics self-efficacy through a social networks lens. *Phys. Rev. Phys. Educ. Res.* 12 1–14. 10.1103/PhysRevPhysEducRes.12.020124

[B25] Dufner M. Reitz A. Zander L. (2015). Antecedents, consequences, and mechanisms: On the longitudinal interplay between academic self-enhancement and psychological adjustment. *J. Pers.* 83 511–522. 10.1111/jopy.12128 25181603

[B26] Dweck C. S. (2000). *Self-Theories: Their Role in Motivation, Personality, and Development.* New York, NY: Psychology Press.

[B27] Ehmke T. Siegle T. (2005). ISEI, ISCED, HOMEPOS, ESCS: Indikatoren der sozialen Herkunft bei der Quantifizierung von sozialen Disparitäten. *Z. Erziehungswiss.* 8 521–539. 10.1007/s11618-005-0157-7

[B28] Else-Quest N. M. Hyde J. S. Linn M. C. (2010). Cross-national patterns of gender differences in mathematics: a meta-analysis. *Psychol. Bull.* 136 103–127. 10.1037/a0018053 20063928

[B29] Else-Quest N. M. Mineo C. C. Higgins A. (2013). Math and science attitudes and achievement at the intersection of gender and ethnicity. *Psychol. Women Q.* 37 293–309. 10.1177/0361684313480694

[B30] Enders C. K. (2010). *Applied Missing Data Analysis.* New York, NY: The Guilford Press.

[B31] Fiedler K. (2014). From intrapsychic to ecological theories in social psychology: outlines of a functional theory approach. *Eur. J. Soc. Psychol.* 44 657–670. 10.1002/ejsp.2069

[B32] Friedman L. (1989). Mathematics and the gender gap: a meta-analysis of recent studies on sex differences in mathematical tasks. *Rev. Educ. Res.* 59 185–213. 10.3102/00346543059002185

[B33] Friedman-Sokuler N. Justman M. (2016). Gender streaming and prior achievement in high school science and mathematics. *Econ. Educ. Rev.* 53 230–253. 10.1016/j.econedurev.2016.04.004

[B34] Galla B. M. Wood J. J. Tsukayama E. Har K. Chiu A. W. Langer D. A. (2014). A longitudinal multilevel model analysis of the within-person and between-person effect of effortful engagement and academic self-efficacy on academic performance. *J. Sch. Psychol.* 52 295–308. 10.1016/j.jsp.2014.04.001 24930821

[B35] Hackett G. Betz N. E. (1981). A self-efficacy approach to the career development of women. *J. Vocat. Behav.* 18 326–339. 10.1016/0001-8791(81)90019-1

[B36] Halpern D. F. Benbow C. P. Geary D. C. Gur R. C. Hyde J. S. Gernsbacher M. A. (2007). The science of sex differences in science and mathematics. *Psychol. Sci. Public Interest* 8 1–51. 10.1111/j.1529-1006.2007.00032.x 25530726 PMC4270278

[B37] Hampton N. Z. (1998). Sources of academic self-efficacy scale: an assessment tool for rehabilitation counselors. *Rehabil. Couns. Bull.* 41 260–277.

[B38] Hanneman R. A. Riddle M. (2011). “Concepts and measures for basic network analysis,” in *The SAGE Handbook of Social Network Analysis*, eds Scott J. Carrington P. J. (Thousand Oaks, CA: SAGE Publications Ltd.), 340–369. 10.4135/9781446294413.n24

[B39] Heatherton T. F. Polivy J. (1991). Development and validation of a scale for measuring state self-esteem. *J. Pers. Soc. Psychol.* 60 895–910. 10.1037/0022-3514.60.6.895

[B40] Hill C. Corbett C. St. Rose A. (2010). *Why so Few? Women in Science, Technology, Engineering and Mathematics.* Washington, DC: AAUW.

[B41] Hox J. van Buuren S. Jolani S. (2015). “Incomplete multilevel data: problems and solutions,” in *Advances in Multilevel Modeling for Educational Research: Addressing Practical Issues Found in Real-World Applications*, eds Harring J. R. Stapleton L. M. Beretvas S. N. (Charlotte, NC: Information Age Publishing Inc), 39–62.

[B42] Huang C. (2013). Gender differences in academic self-efficacy: a meta-analysis. *Eur. J. Psychol. Educ.* 28 1–35. 10.1007/s10212-011-0097-y

[B43] Hyde J. S. (2014). Gender similarities and differences. *Annu. Rev. Psychol.* 65 373–398. 10.1146/annurev-psych-010213-115057 23808917

[B44] Hyde J. S. Fennema E. Lamon S. J. (1990a). Gender differences in mathematics performance: a meta-analysis. *Psychol. Bull.* 107 139–155. 10.1037/0033-2909.107.2.139 2138794

[B45] Hyde J. S. Fennema E. Ryan M. Frost L. A. Hopp C. (1990b). Gender comparisons of mathematics attitudes and affect. *Psychol. Women Q.* 14 299–324. 10.1111/j.1471-6402.1990.tb00022.x

[B46] Hyde J. S. Lindberg S. M. Linn M. C. Ellis A. B. Williams C. C. (2008). Gender similarities characterize math performance. *Science* 321 494–495. 10.1126/science.1160364 18653867

[B47] Hyde J. S. Mertz J. E. (2009). Gender, culture, and mathematics performance. *Proc. Natl. Acad. Sci. U.S.A.* 106 8801–8807. 10.1073/pnas.0901265106 19487665 PMC2689999

[B48] IBM Corp (2017). *IBM SPSS Statistics for Windows, Version 25.0.* Armonk, NY: IBM Corp.

[B49] James W. (1890). *Principles of Psychology, Vol. 1.* New York, NY: Henry Holt.

[B50] Jerusalem M. Satow L. (1999). “Schulbezogene Selbstwirksamkeitserwartung,” in *Skalen zur Erfassung von Lehrer- und Schülermerkmalen. Dokumentation der psychometrischen Verfahren im Rahmen der wissenschaftlichen Begleitung des Modellversuchs Selbstwirksame Schulen*, eds Schwarzer R. Jerusalem M. (Berlin: Humboldt-Universität zu Berlin), 15–16.

[B51] Joët G. Usher E. L. Bressoux P. (2011). Sources of self-efficacy: an investigation of elementary school students in France. *J. Educ. Psychol.* 103 649–663. 10.1037/a0024048

[B52] John O. P. Robins R. W. (1994). Accuracy and bias in self-perception: individual differences in self-enhancement and the role of narcissism. *J. Pers. Soc. Psychol.* 66 206–219. 10.1037/0022-3514.66.1.206 8126650

[B53] Kanny M. A. Sax L. J. Riggers-Piehl T. A. (2014). Investigating forty years of STEM research: how explanations for the gender gap have evolved over time. *J. Women Minor. Sci. Eng.* 20 127–148. 10.1615/JWomenMinorScienEng.2014007246 31502759

[B54] Keller J. (2002). Blatant stereotype threat and women’s math performance: self-handicapping as a strategic means to cope with obtrusive negative performance expectations. *Sex Roles* 47 193–198. 10.1023/A:1021003307511

[B55] Klassen R. M. (2004). A cross-cultural investigation of the efficacy beliefs of South Asian immigrant and Anglo Canadian nonimmigrant early adolescents. *J. Educ. Psychol.* 96 731–742. 10.1037/0022-0663.96.4.731

[B56] Kurman J. (2004). Gender, self-enhancement, and self-regulation of learning behaviors in junior high school. *Sex Roles* 50 725–735. 10.1023/B:SERS.0000027573.36376.69

[B57] Larson L. M. Pesch K. M. Surapaneni S. Bonitz V. S. Wu T.-F. Werbel J. D. (2015). Predicting graduation: the role of mathematics/science self-efficacy. *J. Career Assess.* 23 399–409. 10.1177/1069072714547322

[B58] Lau C. Kitsantas A. Miller A. D. Drogin Rodgers E. B. (2018). Perceived responsibility for learning, self-efficacy, and sources of self-efficacy in mathematics: a study of international baccalaureate primary years programme students. *Soc. Psychol. Educ.* 21 603–620. 10.1007/s11218-018-9431-4

[B59] Legewie J. DiPrete T. A. (2012). *High School Environments, STEM Orientations, and the Gender Gap in Science and Engineering Degrees.* Available online at: https://ssrn.com/abstract=2008733 (accessed April 1, 2020).

[B60] Lent R. W. Lopez F. G. Bieschke K. J. (1991). Mathematics self-efficacy: sources and relation to science-based career choice. *J. Couns. Psychol.* 38 424–430. 10.1037/0022-0167.38.4.424

[B61] Lent R. W. Lopez F. G. Brown S. D. Gore P. A. (1996). Latent structure of the sources of mathematics self-efficacy. *J. Vocat. Behav.* 49 292–308. 10.1006/jvbe.1996.0045 8980086

[B62] Lindberg S. M. Hyde J. S. Petersen J. L. Linn M. C. (2010). New trends in gender and mathematics performance: a meta-analysis. *Psychol. Bull.* 136 1123–1135. 10.1037/a0021276 21038941 PMC3057475

[B63] Matsui T. Matsui K. Ohnishi R. (1990). Mechanisms underlying math self-efficacy learning of college students. *J. Vocat. Behav.* 37 225–238. 10.1016/0001-8791(90)90042-Z

[B64] Multon K. D. Brown S. D. Lent R. W. (1991). Relation of self-efficacy beliefs to academic outcomes: a meta-analytic investigation. *J. Couns. Psychol.* 38 30–38. 10.1037/0022-0167.38.1.30

[B65] Muthén L. K. Muthén B. O. (1998–2012). *Mplus Users’ Guide*, 7th Edn. Los Angeles, CA: Muthén & Muthén. 10.1037/0022-0167.38.1.30

[B66] Neal J. W. Neal Z. P. Cappella E. (2014). I know who my friends are, but do you? Predictors of self-reported and peer-inferred relationships. *Child Dev.* 85 1366–1372. 10.1111/cdev.12194 24320155 PMC4050041

[B67] Nosek B. A. Smyth F. L. (2011). Implicit social cognitions predict sex differences in math engagement and achievement. *Am. Educ. Res. J.* 48 1125–1156. 10.3102/0002831211410683

[B68] OECD (2006). *Women in Scientific Careers. Unleashing the Potential.* Paris: OECD Publishing.

[B69] OECD (2007). *Summary Report of the Workshop on “Women in Science, Engineering and Technology (SET): Strategies for a Global Workforce”.* Paris: OECD Publishing.

[B70] OECD (2012a). *Not a Math Person?* Paris: OECD Publishing.

[B71] OECD (2012b). *PISA 2009 Technical Report.* Paris: OECD Publishing.

[B72] OECD (2014a). *PISA 2012 Results: What Students Know and Can do - Student Performance in Mathematics, Reading and Science (Volume I, Revised Edition).* Paris: OECD Publishing.

[B73] OECD (2014b). *PISA 2012 Technical Report.* Paris: OECD Publishing.

[B74] OECD (2016). *PISA 2015 Results (Volume I). Excellence and Equity in Education.* Paris: OECD Publishing.

[B75] OECD (2017a). *PISA 2015 Technical Report.* Paris: OECD Publishing.

[B76] OECD (2017b). *Report on the Implementation of the OECD Gender Recommendations - Some Progress on Gender Equality but Much Left to do.* Paris: OECD Publishing.

[B77] Pajares F. (1996). Self-efficacy beliefs in academic settings. *Rev. Educ. Res.* 66 543–578. 10.3102/00346543066004543

[B78] Pajares F. (2005). “Gender differences in mathematics self-efficacy beliefs,” in *Gender Differences in Mathematics. An Integrative Psychological Approach*, eds Gallagher A. M. Kaufman J. C. (New York, NY: Cambridge University Press), 294–315. 10.1017/cbo9780511614446.015

[B79] Pajares F. Johnson M. J. Usher E. L. (2007). Sources of writing self-efficacy beliefs of elementary, middle, and high school students. *Res. Teach. English* 42 104–120.

[B80] Parker P. D. Marsh H. W. Ciarrochi J. Marshall S. Abduljabbar A. S. (2014). Juxtaposing math self-efficacy and self-concept as predictors of long-term achievement outcomes. *Educ. Psychol.* 34 29–48. 10.1080/01443410.2013.797339

[B81] Paunonen S. V. Hong R. Y. (2010). Self-efficacy and the prediction of domain-specific cognitive abilities. *J. Pers.* 78 339–360. 10.1111/j.1467-6494.2009.00618.x 20433622

[B82] Phan H. P. (2012). The development of English and mathematics self-efficacy: a latent growth curve analysis. *J. Educ. Res.* 105 196–209. 10.1080/00220671.2011.552132

[B83] Podsakoff P. M. MacKenzie S. B. Lee J.-Y. Podsakoff N. P. (2003). Common method biases in behavioral research: a critical review of the literature and recommended remedies. *J. Appl. Psychol.* 88 879–903. 10.1037/0021-9010.88.5.879 14516251

[B84] Richardson M. Abraham C. Bond R. (2012). Psychological correlates of university students’ academic performance: a systematic review and meta-analysis. *Psychol. Bull.* 138 353–387. 10.1037/a0026838 22352812

[B85] Robins R. W. Beer J. S. (2001). Positive illusions about the self: short-term benefits and long-term costs. *J. Pers. Soc. Psychol.* 80 340–352. 10.1037//0O22-3514.80.2.340 11220450

[B86] Sawtelle V. Brewe E. Kramer L. H. (2012). Exploring the relationship between self-efficacy and retention in introductory physics. *J. Res. Sci. Teach.* 49 1096–1121. 10.1002/tea.21050

[B87] Schafer J. L. Graham J. W. (2002). Missing data: our view of the state of the art. *Psychol. Methods* 7 147–177. 10.1037/1082-989X.7.2.147 12090408

[B88] Schmidt W. H. Jakwerth P. M. McKnight C. C. (1998). Curriculum-sensitive assessment: content does make a difference. *Int. J. Educ. Res.* 29 503–527. 10.1016/S0883-0355(98)00045-7

[B89] Schmidt W. H. Wang H. C. McKnight C. C. (2005). Curriculum coherence: an examination of US mathematics and science content standards from an international perspective. *J. Curriculum Stud.* 37 525–559. 10.1080/0022027042000294682

[B90] Schöber C. Schütte K. Köller O. McElvany N. Gebauer M. M. (2018). Reciprocal effects between self-efficacy and achievement in mathematics and reading. *Learn. Individ. Differ.* 63 1–11. 10.1016/j.lindif.2018.01.008

[B91] Schunk D. H. Ertmer P. A. (2000). “Self-regulation and academic learning. Self-efficacy enhancing interventions,” in *Handbook of Self-Regulation*, eds Boekaerts M. Zeidner M. Pintrich P. R. (Cambridge, MA: Academic Press), 631–649. 10.1016/b978-012109890-2/50048-2

[B92] Snijders T. A. B. Bosker R. J. (2012). *Multilevel Analysis. An Introduction to Basic and Advanced Multilevel Modeling*, 2nd Edn. Los Angeles, CA: SAGE Publications.

[B93] Spencer S. J. Steele C. M. Quinn D. M. (1999). Stereotype threat and women’s math performance. *J. Exp. Soc. Psychol.* 35 4–28. 10.1006/jesp.1998.1373

[B94] Steele C. M. (1997). A threat in the air. How stereotypes shape intellectual identity and performance. *Am. Psychol.* 52 613–629. 10.1037/0003-066X.52.6.613 9174398

[B95] Tiedemann J. (2000). Parents’ gender stereotypes and teachers’ beliefs as predictors of children’s concept of their mathematical ability in elementary school. *J. Educ. Psychol.* 92 144–151. 10.1037//0022-0663.92.U44

[B96] Usher E. L. Pajares F. (2006). Sources of academic and self-regulatory efficacy beliefs of entering middle school students. *Contemp. Educ. Psychol.* 31 125–141. 10.1016/j.cedpsych.2005.03.002

[B97] Usher E. L. Pajares F. (2008). Sources of self-efficacy in school: critical review of the literature and future directions. *Rev. Educ. Res.* 78 751–796. 10.3102/0034654308321456

[B98] Wang M.-T. Kiuru N. Degol J. L. Salmela-Aro K. (2018). Friends, academic achievement, and school engagement during adolescence: a social network approach to peer influence and selection effects. *Learn. Instr.* 58 148–160. 10.1016/j.learninstruc.2018.06.003

[B99] Watermann R. Baumert J. (2006). “Entwicklung eines Strukturmodells zum Zusammenhang zwischen sozialer Herkunft und fachlichen und überfachlichen Kompetenzen. Befunde national und international vergleichender Analysen,” in *Herkunftsbedingte Disparitäten im Bildungswesen. Vertiefende Analysen im Rahmen von PISA 2000*, eds Baumert J. Stanat P. Watermann R. (Wiesbaden: VS Verlag für Sozialwissenschaften), 61–94.

[B100] Wendt H. Bos W. Selter C. Köller O. Schwippert K. Kasper D. (eds). (2016). *TIMSS 2015. Mathematische und Naturwissenschaftliche Kompetenzen von Grundschulkindern in Deutschland im Internationalen Vergleich.* Münster: Waxmann.

[B101] Wentzel K. R. (2017). “Peer relationships, motivation, and academic performance at school,” in *Handbook of Competence and Motivation: Theory and Application*, eds Elliot A. J. Dweck C. S. Yeager D. S. (New York, NY: Guildford Press), 586–603.

[B102] Wigfield A. Eccles J. S. (2000). Expectancy-value theory of achievement motivation. *Contemp. Educ. Psychol.* 25 68–81. 10.1006/ceps.1999.1015 10620382

[B103] Won S. Lee S.-Y. Bong M. (2017). Social persuasion by teachers as a source of student self-efficacy: the moderating role of perceived teacher credibility. *Psychol. Sch.* 54 532–547. 10.1002/pits.22009

[B104] Yeager D. S. Dweck C. S. (2012). Mindsets that promote resilience: when students believe that personal characteristics can be developed. *Educ. Psychol.* 47 302–314. 10.1080/00461520.2012.722805

[B105] Zander L. Brouwer J. Jansen E. Crayen C. Hannover B. (2018). Academic self-efficacy, growth mindsets, and university students’ integration in academic and social support networks. *Learn. Individ. Differ.* 62 98–107. 10.1016/j.lindif.2018.01.012

[B106] Zander L. Kreutzmann M. Hannover B. (2017). Peerbeziehungen im Klassenzimmer. *Z. Erziehwiss.* 20 353–386. 10.1007/s11618-017-0768-9

[B107] Zeldin A. L. Pajares F. (2000). Against the odds: self-efficacy beliefs of women in mathematical, scientific, and technological careers. *Am. Educ. Res. J.* 37 215–246. 10.3102/00028312037001215

