# Supplementary material for: When Grades Are High but Self-Efficacy Is Low: Unpacking the Confidence Gap Between Girls and Boys in Mathematics
Source: Front Psychol. 2020 Oct 7;11:552355. doi: 10.3389/fpsyg.2020.552355 (PMC7580255; doi:10.3389/fpsyg.2020.552355)
Supplement: Supplementary file 1 [file Table_1.pdf]

frontiers

Supplementary Material

1 Measures

Please note that in our study, all measures were administered in German language. For this supplement they were translated to English.

1.1 Mathematics self-efficacy (adapted from Jerusalem & Satow, 1999)

Think of the subject mathematics when indicating your degree of agreement or disagreement with the following statements.

	not true at all	rather not true	neither	rather true	exactly true
If I try hard enough, I can even solve difficult assignments.	☐	☐	☐	☐	☐
In math, it is easy for me to understand new things.	☐	☐	☐	☐	☐
If I had to solve a difficult problem at the blackboard, I believe I would perform well.	☐	☐	☐	☐	☐
Even if I would be sick for a longer period of time, I would perform well.	☐	☐	☐	☐	☐
I am sure I can perform well even if the teacher is doubting my abilities.	☐	☐	☐	☐	☐
If I get a poor grade in math, I am still confident that I can get the grade I want in math.	☐	☐	☐	☐	☐

1.2 Past mastery experience

Which grade did you receive on your last report card in the following subjects?

Mathematics

☐ very good (1)
☐ good (2)
☐ satisfactory (3)
☐ sufficient (4)

- ☐ inadequate (5)
- ☐ insufficient (6)

1.3 Situational mastery experience (example items from TIMSS; Baumert, Bos, Klieme, Lehmann, Lehrke, Hosenfeld, & Neubrand, 1999)

Task 2)

Which of the following mathematical expressions is equivalent to y^3?

- ☐ A. y + y + y
- ☐ B. y · y · y
- ☐ C. 3y
- ☐ D. y^2 + y

Task 6)

This figure is being rotated.

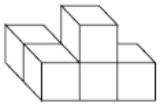

Which of the following figures do you get when rotating the one depicted above?

☐ A.

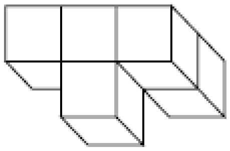

☐ B.

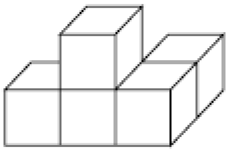

☐ C.

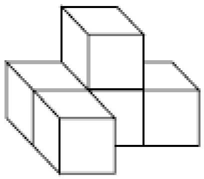

☐ D.

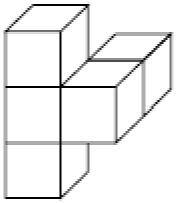

Task 8)

If $\frac{12}{n} = \frac{36}{21}$, then *n is equivalent to*

- ☐ A. 3
- ☐ B. 7
- ☐ C. 36
- ☐ D. 63

Task 11)

Which of the following angles is closest to 30°?

☐ A. ☐ B. ☐ C. ☐ D.

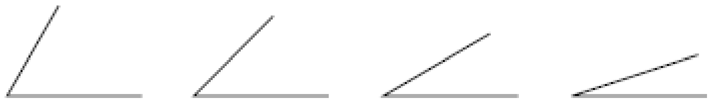

Task 20)

Peter buys 70 pieces of one product, Susi buys 90 pieces. Every piece has the same price. In total, the pieces cost 800 Euro. How much does Susi have to pay?

Answer: Susi pays ________________________

1.4 Situational mastery experience: Cognitive component

What do you think, how many items in the test did you solve correctly? Please make a cross on the line below.

none correct |————————————| all correct

1.5 Situational mastery experience: Affective component (adapted from Heatherton & Polivy, 1991)

How do you feel at the moment?

	not true at all	rather not true	neither	rather true	exactly true
I feel confident about my abilities.	☐	☐	☐	☐	☐
I feel frustrated or rattled about my performance.	☐	☐	☐	☐	☐
I am afraid there is not much I can be proud of right now.	☐	☐	☐	☐	☐
I feel as smart as others.	☐	☐	☐	☐	☐

I feel confident that I understand things.	☐	☐	☐	☐	☐
I feel that I have less scholastic ability right now than others.	☐	☐	☐	☐	☐
I have like I'm not doing well.	☐	☐	☐	☐	☐

1.6 Social persuasion

*Please name the aliases of the persons **that you** would ask for **help** regarding homework in **math**.*		
________________________	________________________	________________________

1.7 Vicarious experiences

For some of the items in the following math test, you will be asked to evaluate your performance and to compare it to the performance of another classmate. You can now choose this person. Please take a look at the list of aliases that your teacher handed out and write down the alias of this student here:

References

Baumert, J., Bos, W., Klieme, E., Lehmann, R., Lehrke, M., Hosenfeld, I., Neubrand, J., & Watermann, R. (Eds.). (1999). *Testaufgaben zu TIMSS/l II Mathematisch-naturwissenschaftliche Grundbildung und voruniversitäre Mathematik und Physik der Abschlußklassen der Sekundarstufe II (Population 3)*. Berlin: Max-Planck-Institut für Bildungsforschung.

Heatherton, T. F., & Polivy, J. (1991). Development and validation of a scale for measuring state self-esteem. *Journal of Personality and Social Psychology, 60*(6), 895-910. doi: 10.1037/0022-3514.60.6.895

Jerusalem, M., & Satow, L. (1999). Schulbezogene Selbstwirksamkeitserwartung. In R. Schwarzer & M. Jerusalem (Eds.), *Skalen zur Erfassung von Lehrer- und Schülermerkmalen. Dokumentation der psychometrischen Verfahren im Rahmen der wissenschaftlichen Begleitung des Modellversuchs Selbstwirksame Schulen* (pp. 15-16). Berlin: Humboldt Universität zu Berlin, Freie Universität Berlin.

2 Supplementary Analyses

Preliminary Analyses

For mathematics self-efficacy, the configural invariance model was a good fit to the data ($\chi2$ = 106.584; *df* = 18; *p* ≤ .001; CFI = .947; RMSEA = .128 (90% CI = .105/.151); SRMR = .041). The fit of the metric invariance was also good ($\chi2$ = 109.626; *df* = 23; *p* ≤ .001; CFI = .948; RMSEA = .112 (90% CI = .091/.133); SRMR = .047), and imposing constraints on the factor loadings did not diminish model fit ($\Delta\chi2$ = 3.042; *df* = 5; *p* = .694). Thus, metric invariance was supported. After adding constraints on item intercepts across gender, overall model fit was still good ($\chi2$ = 117.989; *df* = 28; *p* ≤ .001; CFI = .946; RMSEA = .103 (90% CI = .084/.123); SRMR = .050), and showed no reduction in model fit compared to the metric one ($\Delta\chi2$ = 8.364; *df* = 5; *p* = .137), thereby supporting scalar invariance.

The configural invariance model for state self-esteem was a good fit to the data ($\chi2$ = 105.590; *df* = 28; *p* ≤ .001; CFI = .944; RMSEA = .085 (90% CI = .068/.103); SRMR = .042). The metric invariance model was also a good fit to the data ($\chi2$ = 117.898; *df* = 34; *p* ≤ .001; CFI = .940; RMSEA = .080 (90% CI = .065/.096); SRMR = .056), and showed no significant reduction in model fit compared to the configural one ($\Delta\chi2$ = 12.308; *df* = 6; *p* = .055), thereby supporting metric invariance. After imposing constraints on item intercepts across gender to test for scalar invariance, model fit was still good ($\chi2$ = 135.489; *df* = 40; *p* ≤ .001; CFI = .932; RMSEA = .079 (90% CI = .065/.094); SRMR = .068), but showed a reduction in model fit compared to the metric one ($\Delta\chi2$ = 17.592; *df* = 6; *p* ≤ .01). However, due to its sensitivity to large samples, this test is not fully appropriate for our sample size (cf., Schermelleh-Engel, Moosbrugger, & Müller, 2003). Therefore, we evaluated our data according to Chen (2007): If the CFI does not decrease by more than .010 units (in our case ΔCFI = .008), the RMSEA does not increase by more than .015 units (in our case ΔRMSEA = -0.001), and the SRMR does not increase by .015 units (in our case ΔSRMR = .012) across models, measurement invariance can be assumed. Thus, scalar invariance was supported and the statistical prerequisites for mean value comparisons between both groups were met.

References

Chen, F. F. (2007). Sensitivity of goodness of fit indixes to lack of measurement invariance. *Structural Equation Modeling, 14*(3), 464-504. doi: 10.1080/10705510701301834

Schermelleh-Engel, K., Moosbrugger, H., & Müller, H. (2003). Evaluating the fit of structural equation models: Tests of significance and descriptive goodness-of-fit measures. *Methods of Psychological Research, 8*(2), 23-74.

3 Supplementary Tables

Supplementary Table 1

Regression models predicting students' mathematics self-efficacy for girls and boys

	B (*SE*)				β		χ² (*df*)
	Girls		Boys		Girls	Boys	
**Model 1**							
Test performance[a]	0.015	(0.004)***	0.016	(0.004)***	0.211	0.232	0.038 (1)
**Model 2**							
Test performance[a]	0.004	(0.003)	0.004	(0.004)	0.065	0.064	
Student's mathematics grade[a]	0.414	(0.043)***	0.386	(0.057)***	0.468	0.466	0.300 (2)
**Model 3**							
Test performance[a]	0.015	(0.003)***	0.017	(0.004)***	0.212	0.253	
Self-enhancement score (cognitive)	0.190	(0.047)***	0.267	(0.043)***	0.214	0.310	1.339 (2)
**Model 4**							
Test performance[a]	0.006	(0.004)	0.010	(0.004)**	0.086	0.156	
State self-esteem (affective)[a]	0.423	(0.058)***	0.303	(0.062)***	0.421	0.296	2.466 (2)
**Model 5**							
Test performance[a]	0.009	(0.003)**	0.011	(0.004)**	0.132	0.162	
Social persuasion score[b]	0.205	(0.035)***	0.161	(0.040)***	0.308	0.271	0.912 (2)
**Model 6**							
Test performance[a]	0.014	(0.004)***	0.014	(0.004)***	0.198	0.213	
Mathematics grade CP[a]	0.104	(0.040)**	0.099	(0.045)*	0.117	0.123	0.015 (2)
**Model 7**							
Test performance[a]	-0.001	(0.003)	0.005	(0.004)	-0.011	0.069	
Student's mathematics grade[a]	0.300	(0.053)***	0.295	(0.064)***	0.336	0.356	
Self-enhancement score (cognitive)	0.036	(0.050)	0.174	(0.047)***	0.041	0.202	
State self-esteem (affective)[a]	0.311	(0.068)***	0.115	(0.069)	0.307	0.112	
Social persuasion score[b]	0.057	(0.042)	0.023	(0.041)	0.084	0.038	
Mathematics grade CP[a]	0.012	(0.035)	0.050	(0.036)	0.013	0.062	7.704 (6)

N = 764. All values were estimated using Mplus and full information maximum likelihood (FIML). Standard errors were adjusted for the nesting of students within classes. In the last column, χ^2 differences between a fully unconstrained model and a model assuming equal regression coefficients between girls and boys are indicated. CP = comparison partner. [a] This variable was centered on the group mean. [b] Due to the high skewness of the social persuasion score, ln(score+1) was taken for all analyses. $^{**}p < .01$. $^{***}p < .001$.

Supplementary Table 2

Direct and indirect effects of gender on students' mathematics self-efficacy

	Regression coefficient		Indirect effect of gender on MS via	
	***B* (*SE*)**	**β**	***B* (*SE*)**	**Indirect/Total**
**Model 1**				
Gender	-0.405 (0.066)***	-0.228		
**Model 2**				
Gender	-0.325 (0.067)***	-0.183		
Test performance[a]	0.015 (0.003)***	0.218	-0.074 (0.020)***	18.3%
**Model 3**				
Gender	-0.325 (0.058)***	-0.184		
Test performance[a]	0.005 (0.003)	0.066	-0.022 (0.014)	5.4%
Student's mathematics grade[a]	0.404 (0.037)***	0.463	-0.041 (0.037)	10.1%
**Model 4**				
Gender	-0.228 (0.073)**	-0.128		
Test performance[a]	0.015 (0.003)***	0.225	-0.076 (0.021)***	18.8%
Self-enhancement score (cognitive)	0.223 (0.031)***	0.254	-0.096 (0.020)***	23.7%
**Model 5**				
Gender	-0.214 (0.055)***	-0.122		
Test performance[a]	0.008 (0.003)**	0.115	-0.039 (0.014)**	9.6%
State self-esteem (affective)[a]	0.375 (0.046)***	0.374	-0.141 (0.032)***	34.8%
**Model 6**				
Gender	-0.350 (0.066)***	-0.200		
Test performance[a]	0.010 (0.002)***	0.146	-0.049 (0.015)**	12.1%
Social persuasion score[b]	0.183 (0.030)***	0.285	0.006 (0.020)	-1.5%
**Model 7**				
Gender	-0.319 (0.065)***	-0.180		
Test performance[a]	0.014 (0.003)***	0.203	-0.069 (0.019)***	17.0%
Mathematics grade CP[a]	0.106 (0.033)**	0.121	-0.017 (0.010)	4.2%
**Model 8**				
Gender	-0.180 (0.060)**	-0.104		
Test performance[a]	0.009 (0.003)***	0.133	-0.044 (0.015)**	10.9%
Self-enhancement score (cognitive)	0.108 (0.037)**	0.126	-0.047 (0.017)**	11.6%
State self-esteem (affective)[a]	0.329 (0.054)***	0.332	-0.123 (0.033)***	30.4%

Notes. *N* = 764. All values were estimated using Mplus and full information maximum likelihood (FIML). Standard errors were adjusted for the nesting of students within classes. % Indirect effect is calculated by dividing the specific indirect effect through the total estimated effect of gender on students' mathematics self-efficacy. Gender: 0 = boys, 1 = girls. MS = mathematics self-efficacy; CP = comparison partner. [a] This variable was

centered around the group mean. [b] Due to the high skewness of the social persuasion score, ln(score+1) was taken for all analyses. $^{**}p < .01$. $^{***}p < .001$.
